# Supplementary material for: Mycobacterium tuberculosis RpfE-Induced Prostaglandin E2 in Dendritic Cells Induces Th1/Th17 Cell Differentiation
Source: Int J Mol Sci. 2021 Jul 14;22(14):7535. doi: 10.3390/ijms22147535 (PMC8304802; doi:10.3390/ijms22147535)
Supplement: Supplementary file 1 [file ijms-22-07535-s001.zip › ijms-1263328 - supplementary.pdf]

***Mycobacterium tuberculosis* RpfE-induced prostaglandin E2 in dendritic cells induces Th1/Th17 cell differentiation**

Hye-Soo Park, Seunga Choi, Young Woo Back, Kang-In Lee, Han-Gyu Choi*, Hwa-Jung Kim*

Department of Microbiology, Brain Korea 21 FOUR Project for Medical Science, College of Medicine, Chungnam National University, Daejeon, Republic of Korea

*For correspondence
Hwa-Jung Kim, E-mail: hjukim@cnu.ac.kr,
Han-Gyu Choi, Email: ekdrms20000@cnu.ac.kr

Keywords: tuberculosis, dendritic cell maturation, PGE2, Th1/Th17 differentiation, Naïve T cell differentiation, multifunctional T cell

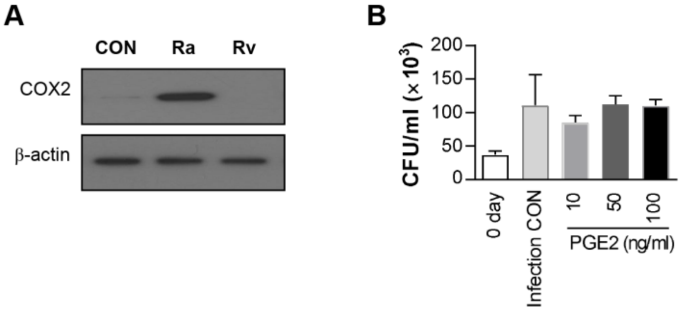

Supplementary Figure 1. Confirmation of prostaglandin E2 (PGE2) production in infected macrophages and the inhibitory effect of PGE2 on *Mycobacterium tuberculosis* (Mtb) growth. (A) The level of COX2 expression in Mtb-infected macrophages was assessed by immunoblotting. (B) Bone marrow-derived macrophages (BMDMs) were treated with PGE2. Intracellular Mtb growth in the BMDMs was determined after 3 days.
